# Genetic determinants of heat resistance in *Escherichia coli*

**DOI:** 10.3389/fmicb.2015.00932

**Published:** 2015-09-09

**Authors:** Ryan G. Mercer, Jinshui Zheng, Rigoberto Garcia-Hernandez, Lifang Ruan, Michael G. Gänzle, Lynn M. McMullen

**Affiliations:** ^1^Department of Agricultural, Food and Nutritional Science, University of AlbertaEdmonton, AB, Canada; ^2^State Key Laboratory of Agricultural Microbiology, Huazhong Agricultural UniversityWuhan, China

**Keywords:** STEC, VTEC, EHEC, O157, heat resistance, beef, Cronobacter, Klebsiella

## Abstract

*Escherichia coli* AW1.7 is a heat resistant food isolate and the occurrence of pathogenic strains with comparable heat resistance may pose a risk to food safety. To identify the genetic determinants of heat resistance, 29 strains of *E. coli* that differed in their of heat resistance were analyzed by comparative genomics. Strains were classified as highly heat resistant strains, exhibiting a D_60_-value of more than 6 min; moderately heat resistant strains, exhibiting a D_60_-value of more than 1 min; or as heat sensitive. A ~14 kb genomic island containing 16 predicted open reading frames encoding putative heat shock proteins and proteases was identified only in highly heat resistant strains. The genomic island was termed the locus of heat resistance (LHR). This putative operon is flanked by mobile elements and possesses >99% sequence identity to genomic islands contributing to heat resistance in *Cronobacter sakazakii* and *Klebsiella pneumoniae*. An additional 41 LHR sequences with >87% sequence identity were identified in 11 different species of β- and γ-proteobacteria. Cloning of the full length LHR conferred high heat resistance to the heat sensitive *E. coli* AW1.7ΔpHR1 and DH5α. The presence of the LHR correlates perfectly to heat resistance in several species of *Enterobacteriaceae* and occurs at a frequency of 2% of all *E. coli* genomes, including pathogenic strains. This study suggests the LHR has been laterally exchanged among the β- and γ-proteobacteria and is a reliable indicator of high heat resistance in *E. coli*.

## Introduction

*Escherichia coli* are commensals in the human and animal gut but the species also comprises intestinal and extraintestinal pathogens. The ecological versatility of *E. coli* is reflected in its genome plasticity. The average *E. coli* genome is approximately 5.16 Mb, encoding an average of 5190 genes. The core genome of *E. coli* comprises about 1700 genes (Kaas et al., 2012); however, the pan-genome of *E. coli* contains more than 18,000 genes and is still considered to be open (Rasko et al., 2008; Touchon et al., 2009; Kaas et al., 2012).

Lateral gene transfer promotes the evolution and diversity of *E. coli*, and allows acquisition of virulence factors (Dobrindt et al., 2004; Croxen et al., 2013; Gordienko et al., 2013). Genes responsible for colonization, toxin production and antibiotic resistance are encoded on mobile genetic elements and are transmitted between strains of *E. coli* (Croxen et al., 2013). One prominent example is the Shiga toxin (*stx1* or *stx2*), carried on the genomes of lambdoid prophages (O'Brien et al., 1984). The horizontal transfer of large gene clusters, called genomic islands, also provides accessory genes for niche adaptation and pathogenicity (reviewed in Schmidt and Hensel, 2004; Rasko et al., 2008; Croxen et al., 2013). The locus of enterocyte effacement (LEE) is a 35-kb genomic island coding for virulence genes for attachment and effacement of intestinal epithelial cells and other pathogenic traits (McDaniel et al., 1995). Novel combinations of accessory genes present significant challenges to public health. Transduction of an *E. coli* by a Shiga toxin-converting phage resulted in a new pathovar, enteroaggregative hemorrhagic *E. coli*, which caused a large foodborne outbreak in summer 2011 (Bielaszewska et al., 2011).

Pathovars of *E. coli* are characterized by their virulence gene profile, mechanisms for cellular adhesions, and site of colonization, and include enteropathogenic *E. coli* (EPEC), enterohemorrhagic *E. coli* (EHEC), enteroaggregative *E. coli* (EAEC), enteroaggregative hemorrhagic *E. coli* (EAHEC), enterotoxigenic *E. coli* (ETEC), and uropathogenic *E. coli* (UPEC) (Agarwal et al., 2012; Croxen et al., 2013). Due to the severity of infections and the low infectious dose, EHEC and EAHEC are particularly of concern for both public health and the food industry (Bielaszewska et al., 2011; Scallan et al., 2011; Croxen et al., 2013). EHEC carry *stx* genes and are also referred to as Shiga toxin-producing *E. coli* (STEC) (Croxen et al., 2013). STEC causes an estimated 175,000 food-borne infections per year in the United States (Scallan et al., 2011). The most frequent serotype of STEC in North America is O157:H7, but other serotypes have also been implicated in STEC infections and are food adulterants in the U.S. (USDA, 2012).

Ruminants including cattle are the primary reservoir for STEC (Lainhart et al., 2009; Croxen et al., 2013). The beef processing industry applies thermal intervention methods such as steam pasteurization and hot water washes to reduce STEC contamination of meat. However, heat resistance in *E. coli* is highly variable and some strains exhibit a stable thermotolerant phenotype (Rudolph and Gebendorfer, 2010). While most strains of *E. coli* have D_60_ values of less than 1 min, moderately or exceptionally heat resistant strains exhibit D_60_ values of more than 1 and more than 10 min, respectively (Hauben et al., 1997; Dlusskaya et al., 2011; Liu et al., 2015). The beef isolate *E. coli* AW1.7 has a D_60_ value of more than 60 min and survives in beef patties grilled to a core temperature of 71°C or “well done” (Dlusskaya et al., 2011). Heat resistance in *E. coli* AW1.7 has been attributed to the accumulation of compatible solutes (Pleitner et al., 2012) and membrane transport proteins including the outer membrane porin NmpC (Ruan et al., 2011); however, the genetic determinants of heat resistance remain unknown. This study aimed to identify genetic determinants of heat resistance in *E. coli* by comparative genomic analysis of heat sensitive, moderately heat resistant, and extremely heat resistant strains of *E. coli*. Analyses focused on food and clinical isolates of *E. coli* and included Shiga-toxin producing strains.

## Materials and methods

### Strain selection and heat treatments

The 29 strains of *E. coli* used in this study included previously characterized heat resistant and sensitive food and clinical isolates (Ruan et al., 2011; Liu et al., 2015). Strains were selected to include isolates differing in their heat resistance, and to include the phylogenetic variability in the species *E. coli*. All strains were grown overnight in Luria-Bertani (LB) broth at 37°C with 200 rpm agitation. To determine the heat resistance, *E. coli* strains were treated at 60°C for 5 min as previously described (Dlusskaya et al., 2011). After heating, the cultures were serially diluted, plated onto LB agar and incubated aerobically overnight at 37°C. Strains were classified into phenotypic groups based on their survival after heating. Strains with a reduction in cell counts of more than 5 log (cfu mL^−1^) after 5 min at 60°C were classified as heat sensitive. Strains demonstrating a reduction in cell counts of 1 to 5 log (cfu mL^−1^) were classified as moderately heat resistant while strains with reductions less than 1 log (cfu mL^−1^) designated as highly heat resistant.

### Genomic DNA isolation, genome sequencing, assembly, and annotation

DNA for genome sequencing was isolated from overnight cultures of *E. coli* grown in 5 ml of LB broth. Genomic DNA was isolated using the Wizard® Genomic DNA Purification Kit (Promega, Madisson, Wisconsin, USA) following the manufacturer's guidelines. The quality and quantity of each sample was assessed using gel electrophoresis and a NanoDrop® 2000c spectrophotometer (Thermo Scientific, Wilmington, Delaware, USA). DNA samples were sequenced using Illumina HiSeq2000 with an insert size of 300 bp by Axeq Technologies (Seoul, South Korea). The quality of the 100-bp paired-end reads was assessed using the FastQC tool (http://www.bioinformatics.bbsrc.ac.uk/projects/fastqc) and low quality reads were filtered by Quake (Kelley et al., 2010). Assemblies were obtained using ABySS 1.3.4 (Assembly By Short Sequence; Simpson et al., 2009) with the most optimal k-mer value for each genome. Genomes were annotated automatically by the RAST server. For O157:H7 strains, the genomes assemblies were improved by scaffolding the contigs using the reference genomes of strains EDL933 (Accession: NC002655) and Sakai (Accession: NC002695). All genomic sequences of the 29 strains used in this current study are deposited to the NCBI wgs database under BioProject PRJNA277539.

### Core genome phylogenetic analysis and identification of orthologous genes unique to different phenotypes

To construct a core-genome phylogenetic tree, the 28 sequenced genomes obtained in this study were combined with 48 reference genomes obtained from NCBI Genbank (ftp://ftp.ncbi.nlm.nih.gov/genome) for a total of 76 *E. coli* and *Shigella* genomes. Reference genomes were selected to prioritize closed genomes over whole genome shotgun sequences, and to represent the phylogroups A, B1, B2, D, E, and *Shigella*. Construction of the core genome phylogenetic tree employed the previously described workflow (Touchon et al., 2009; Hazen et al., 2013) including genome alignment to identify the core genome, extraction of nucleotide sequences of the core genome, and calculation of a maximum likelihood phylogenetic tree. The genomes were aligned with Mugsy with default parameters (Angiuoli and Salzberg, 2011). Homologous blocks present in each genome were extracted and concatenated using an in-house Perl script. The most disordered regions were eliminated using Gblocks with default parameters (Talavera and Castresana, 2007). The core genome size of the 76 genomes was approximately 2.7 Mbp. A maximum likelihood phylogenetic tree was constructed by RaxML with default parameters (Stamatakis, 2014) using bootstrapping for 1000 replicates.

To identify orthologous genes unique to the different phenotypic groups, protein sequences from all 29 genomes were combined and searched using all-against-all BLAST. The protein sequences with identities and coverage above 70% were clustered into families using the program OrthoMCL (Li et al., 2003). The inflation value of 2 was used for the MCL clustering. Sequence identity and comparisons of open reading frames (ORFs) were analyzed in Geneious (Biomatters, Auckland, New Zealand).

For phylogenetic analysis of the locus of heat resistance, genomes containing homologous sequences with >80% coverage of the ~14 kb LHR nucleotide sequence from *E. coli* AW1.7 were retrieved from NCBI. Sequences with homology to the LHR of *E. coli* AW1.7 were manually extracted and aligned with ClustalW implemented in MEGA6 (Tamura et al., 2013). The MEGA6 oftware package was used to construct a maximum-likelihood tree with default parameters. Bootstrap support values were calculated from 100 replicates.

To assess frequency of the locus of heat resistance in *E. coli*, a BLAST search of both the NCBI Genomes and whole-genome shotgun assemblies (wgs) database was performed. For each study, the number of strains containing sequence corresponding to >80% coverage was counted and totaled to give an approximate percentage of strains that were positive for the locus. Bioinformatic characterization of the genetic island was completed using BPROM (Solovyev and Salamov, 2011) and ARNold (Gautheret and Lambert, 2001) to identify putative promoters and rho-independent terminator sequences, respectively.

### Genetic complementation of the LHR

To construct a plasmid-borne copy of the LHR, primers were designed in Geneious to selectively amplify the entire genomic island in 3 separate fragments. All primers and plasmids used in this study are listed in Table 1. PCR reactions were carried out using Phusion High-Fidelity DNA polymerase (Thermo Scientific) according to manufacturer guidelines. F1 (~6 kb) was amplified using primer pair HR-R1/HR-R1 and included the native putative promoter sequence. F2 (~3.3 kb) and F3 (~7 kb) were amplified by primer pairs HR-F2.1/HR-R2 and HR-F3/HR-R3, respectively. F1 and F2 were cloned separately into pUC19 as KpnI/XbaI inserts, while F3 was inserted as a XbaI/HindIII fragment, yielding recombinant vectors pUCF1, pUCF2, and pUCF3 (Table 1). All 3 fragments were sequenced and subsequently sub-cloned into the low-copy plasmid, pRK767 (Gill and Warren, 1988), generating pRF1, pRF2, and pRF3 (Table 1). To construct the entire LHR on a plasmid, F1 was ligated into pUCF2 as a KpnI/SmaI fragment, resulting in pUCF1-2. The 8.3 kb insert, F1-2, was sub-cloned to pRK767 as a KpnI/XbaI fragment. The new recombinant vector, pRF1-2, and F3 were digested with BglII and HindIII and ligated to form pRF1-2-3, or simply, pLHR. The plasmids pRF1, pRF2, pRF3, and pLHR were electroporated into *E. coli* AW1.7ΔpHR1, a heat sensitive derivative of AW1.7 (Pleitner et al., 2012). The resulting strains, as well as the DH5α strains used for cloning, were assayed for heat resistance as previously described (Liu et al., 2015). All transformants carrying either pUC19- or pRK767-based recombinant vectors were plated on LB media containing 50 mg l^−1^ ampicillin or 15 mg l^−1^ tetracycline, respectively.

**Table 1:** **Primers and plasmids used in this study**.

**Primer**	**Sequence (5′ → 3′)**	**References**
HR-F1	TTAGGTACCGCTGTCCATTGCCTGA	This study
HS-R1	AGACCAATCAGGAAATGCTCTGGACC	This study
HR-R1	TATCTAGAGTCGCGTGCCAATACCAGTTC	This study
HR-F2.1	AGGGTACCAGCGATATCCGTCAATTGACT	This study
HR-F2.2	GAGGTACCTGTCTTGCCTGACAACGTTG	This study
HR-R2	TATCTAGAATGTCATTTCTATGGAGGCATGAATCG	This study
HR-F3	TATCTAGAGATGGTCAGCGCAGCG	This study
HS-F1	GCAATCCTTTGCCGCAGCTATT	This study
HR-R3	GTCAAGCTTCTAGGGCTCGTAGTTCG	This study
**Plasmids**	**Description**	**References**
pUC19	High-copy cloning vector	Sigma
pRK767	Low-copy cloning vector	Gill and Warren, 1988
pUCF1	LHR fragment 1 in pUC19	This study
pUCF2	LHR fragment 2 in pUC19	This study
pUCF3	LHR fragment 3 in pUC19	This study
pUCF1-2	LHR fragments 1-2 in pUC19	This study
pRF1	LHR fragment 1 in pRK767	This study
pRF2	LHR fragment 2 in pRK767	This study
pRF3	LHR fragment 3 in pRK767	This study
pRF1-2	LHR fragments F1-2 in pRK767	This study
pLHR	The entire LHR sequence, F1-F2-F3, in pRK767	This study

### PCR screening of beef isolates for heat resistance

A set of 55 strains of *E. coli* that were previously isolated from a beef processing plant (Dlusskaya et al., 2011) was screened for heat resistance with primers targeting the locus of heat resistance. *E. coli* AW1.7 and its heat sensitive derivative *E. coli* AW1.7ΔpHR1 (Pleitner et al., 2012) were used as positive and negative controls, respectively. Primers (Table 1) were designed and used to selectively target 3 separate regions (size ranging 1.8–2.8 kb) of the locus of heat resistance. The primer pairs HR-F1/HS-R1 and HR-F2.2/HR-R2 amplified regions A (~1.8 kb) and B (~2.8 kb), respectively. Primers HS-F1 and HR-R3 were used to amplify region C (~2.8 kb). Recombinant Taq® DNA polymerase (Invitrogen, Burlington, Ontario) was used to amplify products in a standard colony PCR reaction mixture and amplicons were visualized by staining with SYBRsafe (Invitrogen, Burlington, Ontario) after agarose gel electrophoresis. Heat resistance for strains *E. coli* MB1.8, DM19.2, AW1.1, GM12.6, MB 16.6, MB 3.5, GM9.1, and AW 12.2 (Dlusskaya et al., 2011) was determined by incubation at 60°C for 5 min and enumeration of surviving cells as described above.

## Results

### Heat resistance of *E. coli*

**Figure 1:**
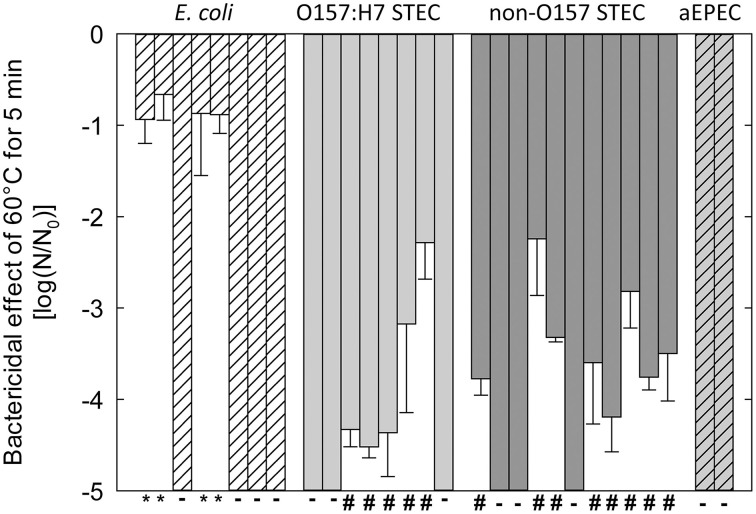
**Reduction of cell counts of strains of *E. coli* after heating at 60°C for 5 min in LB broth**. Strains are separated based on pathotypes: no virulence factors (*E. coli*); O157:H7 STEC and non-O157 STEC); *eae*^+^ *stx*^−^ (atypical EPEC or aEPEC). **-** indicates strains that were designated as “heat sensitive” because cell counts were reduced by more than 5 log (cfu mL^−1^). ^#^Indicates strains that were designated as “moderately heat resistant” because the reduction of cell counts was less than 5 log (cfu mL^−1^). ^*^Indicates strains that were designated as “highly heat resistant” because the reduction of cell counts was less than 1 log (cfu mL^−1^). The figure includes data from Liu et al. (2015).

Strains of *E. coli* were selected for genome sequencing to obtain a wide range of heat resistance despite the limited number of strains (Figure 1). In Figure 1, strains are grouped based on their virulence profiles. O157:H7 STEC and non-O157 STEC were grouped based on serotype and the presence of *stx1* or *stx2* coding for Shiga toxins (Liu et al., 2015). Strains from four groups included heat sensitive strains (Figure 1), in agreement with the heat sensitivity of a majority of strains of *E. coli* (Hauben et al., 1997; Dlusskaya et al., 2011; Liu et al., 2015). Both O157:H7 STEC and non-O157:H7 STEC included moderately heat resistant strains (Figure 1). Four non-pathogenic strains of *E. coli* including *E. coli* AW1.7 were highly heat resistant. All of these strains were obtained from a beef processing facility (Dlusskaya et al., 2011).

### Genome sequences and characteristics

The 29 *E. coli* genomes sequences obtained in this study included genomes from 4 highly heat resistant strains, 13 moderately resistant strains, and 12 heat sensitive strains (Figure 1, Table 2). Genebank accession numbers of the 29 genomes sequenced in this study are indicated in Table 2. The number of contigs larger than 500 bp per genome ranged from 95 to 277, with max sequence lengths ranging from 204263–435416 bp (Table 2).

**Table 2:** ***E. coli* strain used in this study and features of their genome sequences**.

**Accession Numbers**	**Strain (references); phylogenetic group**	**Coverage (X)^a^**	**Number of contigs assembled**	**Max contig size (bp)**	**Number of putative proteins^b^**	**Heat Resistance**	**EHEC Virulence Factors**	**Origin**
LDYI00000000	AW1.3 (1); A	579.12	184	317424	5041	High	n.d.^c^	Beef
LDYL00000000	DM18-3 (2); A	469.26	111	353387	4700	High	n.d.	Beef
LDYM00000000	GM16-6 (2); A	442.89	164	209077	4678	High	n.d.	Beef
LDYJ00000000	AW1.7 (1); A	494.63	165	245564	4971	High (1)	n.d.	Beef
LDYK00000000	AW1.7ΔpHR1; A	519.61	152	246187	4952	Sensitive (3)	n.d.	AW1.7 mutant
LECO00000000	O103:H2 PARC444 (2); B1	471.64	98	356993	4864	Sensitive (2)	n.d.	Unknown
LECG00000000	O103:H2 PARC445 (2); B1	584.81	158	327869	5096	Sensitive (2)	n.d.	Unknown
LECL00000000	O44:H18 PARC450 (2); E	458.44	146	343811	4951	Sensitive (2)	n.d.	Unknown
LEAF00000000	O157:H7 CO6CE1353 (2); D	484.64	205	376588	5572	Moderate (2)	*stx1 stx2 eae*	Clinical
LEAG00000000	O157:H7 CO6CE1943 (2); D	477.76	185	374853	5436	Moderate (2)	*stx1 stx2 eae*	Clinical
LEAH00000000	O157:H7 CO6CE2940 (2); D	475.57	197	376618	5537	Moderate (2)	*stx2 eae*	Clinical
LEAE00000000	O157:H7 CO6CE900 (2); D	470.23	225	376513	5554	Moderate (2)	*stx2 eae*	Clinical
LEAJ00000000	O157:H7 E0122 (2); D	480.56	189	399998	5478	Moderate (2)	*stx2 eae*	Cattle
LEAD00000000	O157:H7 1935 (2); D	502.88	194	393069	5523	Sensitive (2)	*stx1 stx2 eae*	Human
LEAI00000000	O157:H7 CO283 (2); D	531.16	184	376583	5296	Sensitive (2)	*stx1 stx2 eae*	Cattle
LEAK00000000	O157:H7 LCDC7236 (2); D	492.65	181	376583	5461	Sensitive (2)	*stx1 stx2 eae*	Human
LDYN00000000	O26:H11 05-6544 (2)	426.65	280	219684	5691	Moderate (2)	*stx1 eae*	Human
LECF00000000	O103:H25 338 (2); B1	439.31	218	376897	5321	Moderate (2)	*stx1 eae*	Clinical
LECH00000000	O104:H4 11-3088 (2); B1	515.77	173	320350	5254	Moderate (2)	*stx2*^d^	Human
LECI00000000	O111:NM 583 (2); B1	492.35	185	323305	5067	Moderate (2)	*stx1 eae*	Clinical
LECK00000000	O113:H4 09-0525 (2); A	475.86	165	254878	5275	Moderate (2)	*stx1 stx2*	Unknown
LDZZ00000000	O121:H19 03-2832 (2); B1	457.58	213	434838	5272	Moderate (2)	*stx2 eae*	Human
LEAA00000000	O121:NM 03-4064 (2); B1	568.02	221	435416	5298	Moderate (2)	*stx2 eae*	Human
LEAB00000000	O145:NM 03-6430 (2); n.a.	528.20	210	359240	5371	Moderate (2)	*stx1 eae*	Human
LECM00000000	O45:H2 05-6545 (2); B1	508.74	263	261384	5352	Sensitive (2)	*stx1 eae*	Human
LECN00000000	O76:H19 09-0523 (2); B1	456.09	191	404223	5432	Sensitive (2)	*stx1 stx2*	Unknown
LECJ00000000	O111:NM PARC447 (2); B1	544.42	200	376589	5672	Sensitive (2)	*stx1 stx2 eae*	Unknown
LDYO00000000	O26:H11 PARC448; B1	489.45	240	204263	5429	Sensitive (2)	*eae*	Unknown
LEAC00000000	O145:NM PARC449 (2); n.a.	502.50	181	328848	5390	Sensitive (2)	*eae*	Unknown

n.a., not assigned;

a Based on the Lander-Waterman equation using an average size of E. coli genome (5.16 Mb);

b Based on OrthoMCL analysis of all annotated genes;

c n.d., not detected;

d Carries at least the beta lactamase gene present on pHUSEC2011-2 present in EAEC. Other genes on this plasmid includes factors for adhesion.

References: (1) Dlusskaya et al., 2011; (2), Liu et al., 2015; (3), Pleitner et al., 2012.

Genome sequence data confirmed the presence or absence of *stx1/stx2* and *eae* that was determined earlier by PCR (Liu et al., 2015, Table 2). The atypical EPEC (aEPEC) carried the *eae* gene, but no pEAF-encoded *bfp* (bundle-forming pilus) genes (Trabulsi et al., 2002). Other strains of *E. coli* were negative for *eae, stx1/2* and *bfp*. None of the highly heat resistant strains of *E. coli* carried any virulence factors (Table 2). The genomes of *E. coli* AW1.7 and its heat-sensitive derivative *E. coli* AW1.7ΔpHR1 (Pleitner et al., 2012) were virtually identical; however, in addition to the loss of the 4842 bp plasmid pHR1, two additional deletions of 21768 and 16248 bp were identified in the heat sensitive *E. coli* AW1.7ΔpHR1. Of the 19 STEC, 16 possessed the *eae* gene/LEE pathogenicity island; the remaining 3 STEC were categorized as LEE negative STECs (Table 2), which still have the ability to cause disease (Newton et al., 2009). The 19 STEC included moderately heat resistant and heat sensitive strains (Table 2). A moderately heat resistant STEC isolate from the 2011 Germany outbreak, O104:H4 11-3088, carried *stx2*, as well as a gene encoding a β-lactamase from the EHEC plasmid, pHUSEC2011. This plasmid also encodes EAEC virulence factors such as the *aaf* and *agg* genes (Estrada-Garcia and Navarro-Garcia, 2012).

### Phylogenetic distribution of heat resistant isolates

**Figure 2:**
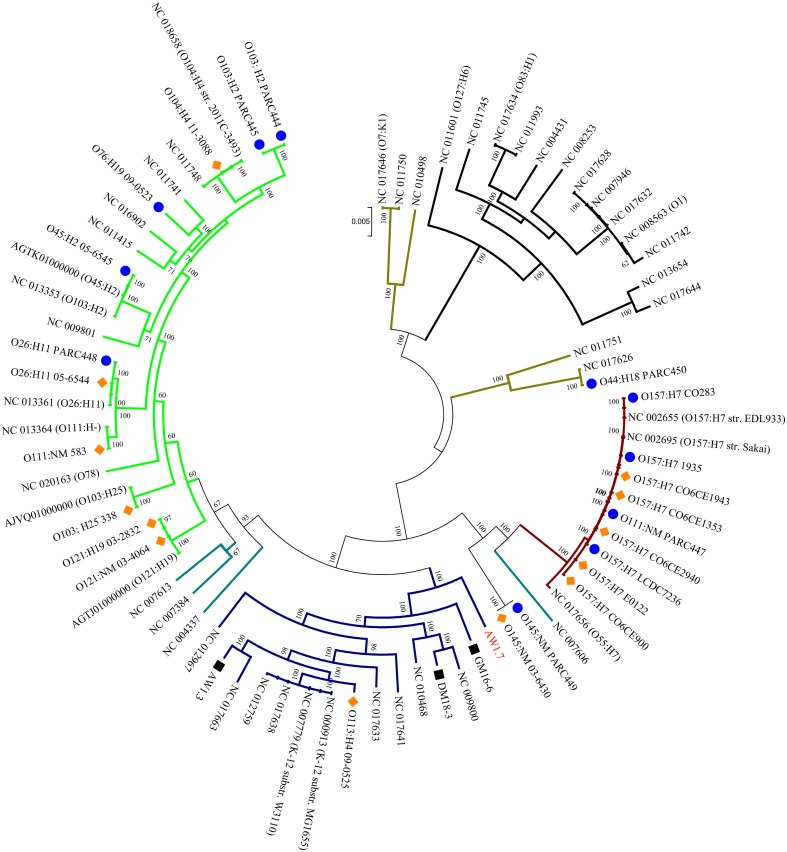
**Phylogenomic distribution of strains of *E. coli* and *Shigella* spp**. A core genome phylogenetic tree was constructed using the 28 sequenced genomes from this project, indicated by strain numbers and serotype as applicable, and 48 genome sequences from NCBI, indicated by serotype and Accession numbers. The strain numbers of the 48 strains are indicated in Table S2. The phylogenetic groups of *E. coli* are color coded: A, Blue; B1, Green; B2, Black; D, Brown; and E, Maroon; *Shigella* spp. (indicated by teal colored branches) were included in the phylogenetic tree because this genus is considered a host-adapted pathovar of *E. coli*. Bootstrapping values are indicated for each branch. The sequenced genomes from this project are coded by blue circles and orange diamonds indicating heat sensitive and moderately heat resistant strains, respectively. Black squares represent highly heat resistant strains.

To assess the phylogenetic relationships of the heat resistant and sensitive strains, a core genome phylogenetic tree was constructed with the genomes from this study, and 48 obtained from the NCBI database. The *E. coli* phylogenetic groups A, B1, B2, D and E (Jaureguy et al., 2008; Touchon et al., 2009) were well supported by our core genome tree (Figure 2).

Moderately heat resistant strains were found in the phylogenetic groups A, B1, and E (Figure 2). Resistant and sensitive strains of the serotype O157H7 and O26:H11, 05-6544 and PARC448, respectively, group together near NCBI strains of similar serotypes. This grouping of heat resistant and sensitive isolates occurs with O145:NM isolates as well, however, these strains are found distinctly separate from other phylogenetic groups (Figure 2). Some moderately resistant, non-O157 STEC are located on branches with pathogenic *E. coli* including O104:H4 11-3088 (Figure 2). The overall genomic similarity of sensitive and resistant strains may illustrate the ease of acquiring genetic variations to become moderately heat resistant. Particularly strains within phylogenetic group E, comprising O157:H7 STEC (Figure 2), are highly related and therefore the differences in the accessory genes, content or sequence, accounts for differences in heat resistance.

All four highly resistant strains were assigned to group A. The highly heat resistant strains *E. coli* AW1.7 and GM16.6 are located in divergent branches separate from other *E. coli* in this group (Figure 2). *E. coli* AW1.3 shares a high degree of sequence similarity to *E. coli* P12b, a model strain for flagellar studies (Ratiner et al., 2010), while *E. coli* DM18.3 is closely related to the commensal *E. coli* strain HS (Levine et al., 1978). The phylogenetic diversity of highly heat resistant strains indicates that these strains do not share a common ancestor (Figure 2).

### Identification the locus of heat resistance (LHR)

**Figure 3:**
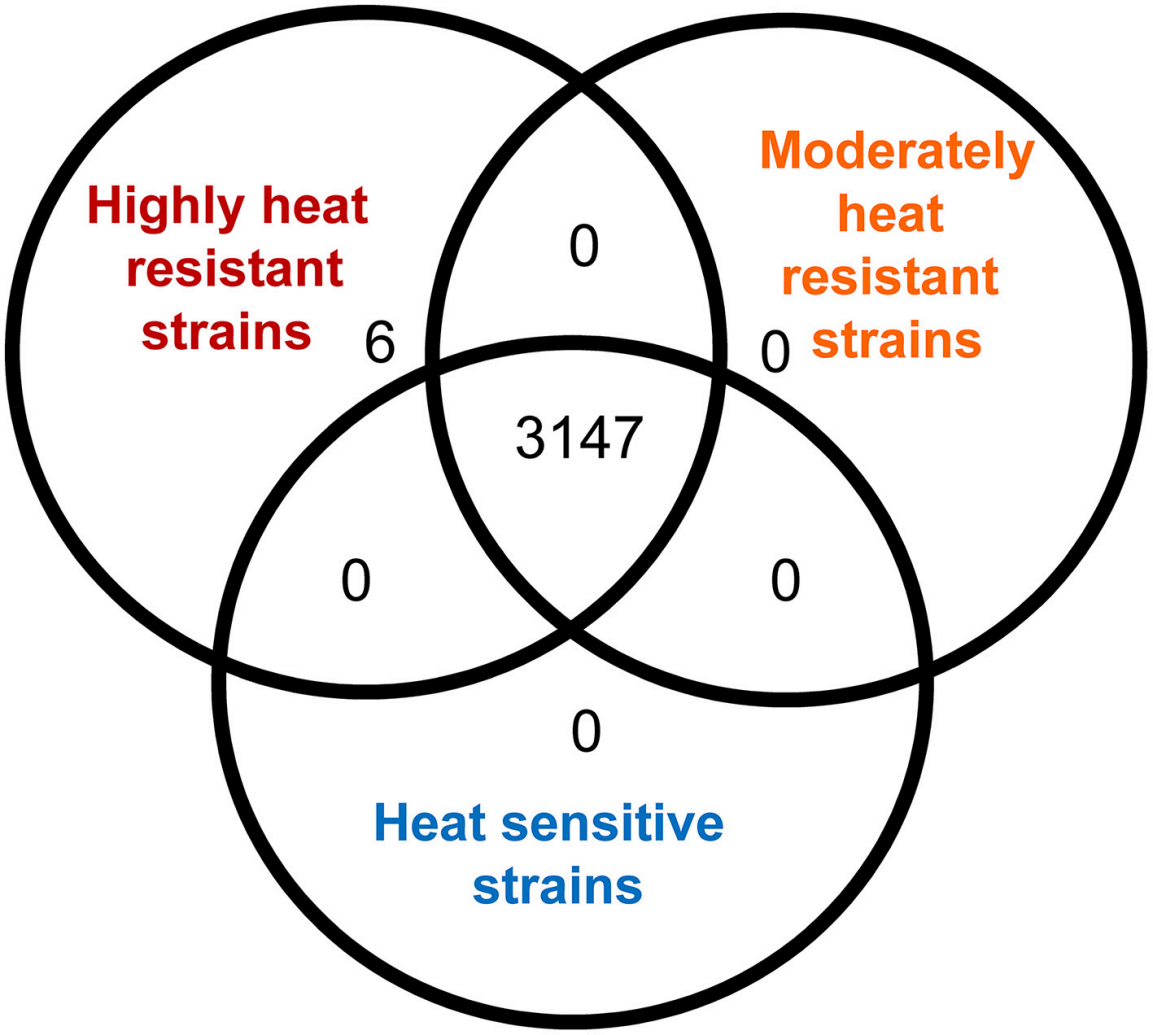
**Analysis of orthologous protein coding sequences identified in highly heat resistant, moderately heat resistant and heat sensitive *E. coli* strains by OrthoMCL**. The Venn diagram indicates the number of protein coding sequences that are shared by all strains analysed in this study, the number of protein coding sequences that were shared between any two of the phenotypic groups, and the number of protein coding sequences that were found only in one of the three phenotypic groups.

**Figure 4:**
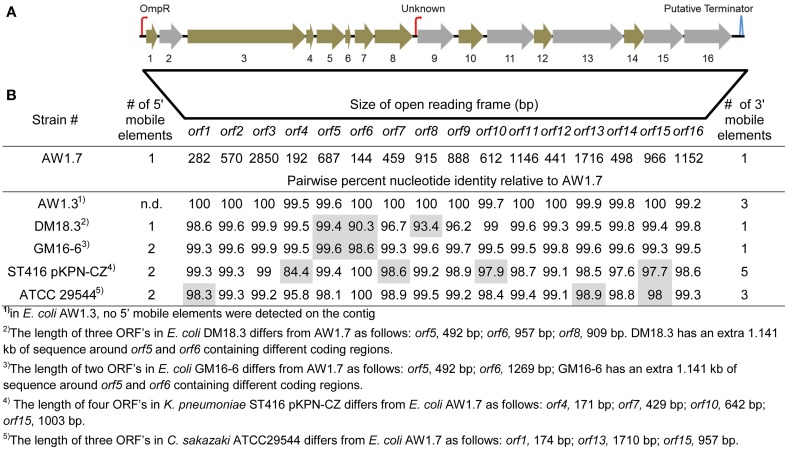
**Representation of the locus of heat resistance (LHR) in *E. coli* AW1.7, AW1.3, DM18.3, and GM16-6, *K. pneumoniae* ST416 pKPN-CZ and *C. sakazaki* ATCC29544**. **(A)** Representation of the LHR in highly heat resistant strains. The figure is scaled to the locus of heat resistance in *E. coli* AW1.7 (14.469 kb in size). Putative promoters and terminators sequences are indicated with hooked arrows and stem-loops, respectively. Open reading frames (ORFs) shaded in gray were identified as unique orthologs in highly heat resistant strains. The GC content of the genetic island is 61.8% while the genome average for AW1.7 is 51.1%. **(B)** Pairwise nucleotide identity of ORFs in *E. coli* AW1.3, DM18.3, and GM16-6, *K. pneumoniae* ST416 pKPN-CZ and *C. sakazaki* ATCC29544 to the corresponding ORFs in *E. coli* AW1.7. ORFs that differ in size from *E. coli* AW1.7 are shaded in gray and the size is indicated in footnotes. Mobile genetic elements were detected in all strains upstream and downstream of the locus of heat resistance; the number of mobile genetic elements is also indicated.

To identify differences in gene content conferring high heat resistance, the genomes were separated into their phenotypic groups: highly resistant; moderately resistant; and sensitive. An OrthoMCL analysis found 3147 orthologs shared among all 28 genomes, however, none of these were unique to heat sensitive or moderately heat resistant strains (Figure 3). A set of 6 genes was unique to the highly heat resistant strains (Figure 3); all of these genes are located on a 14,469 bp genomic island present in *E. coli* AW1.7, AW1.3, DM18.3, and GM16.6 (Figure 4). The 6 genes specific to the highly heat resistant group are scattered among an additional 10 ORFs in this genomic island (Figure 4). Remarkably, this genomic island was absent in *E. coli* AW1.7ΔpHR1. The plasmid curing protocol used to generate *E. coli* AW1.7ΔpHR1 (Pleitner et al., 2012) thus also resulted in a 16,248 bp deletion encompassing the genomic island and the flanking mobile genetic elements. This operon was previously identified in heat resistant strains of *Cronobacter sakazakii* (Gajdosova et al., 2011) and *Klebsiella pneumoniae* (Bojer et al., 2010). Due to its presence in highly heat resistant *E. coli*, the genomic island was named the locus of heat resistance (LHR).

### LHR confers high heat resistance to heat sensitive *E. coli*

**Figure 5:**
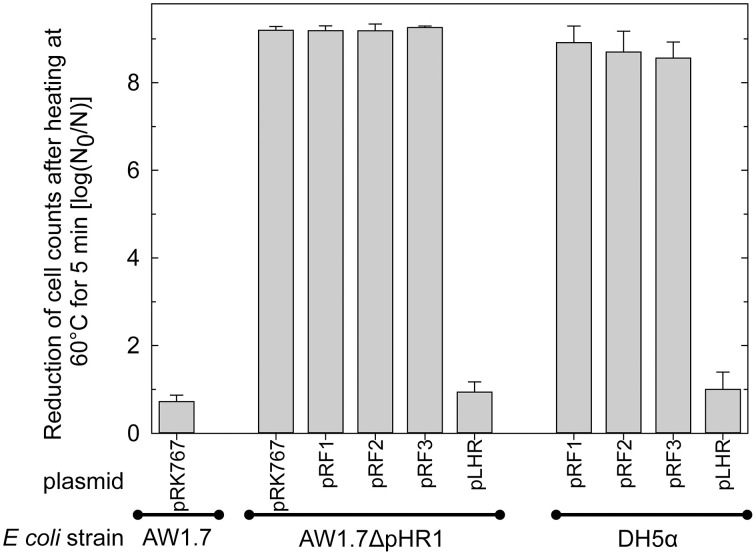
**Heat resistance of *E. coli* AW1.7, AW1.7ΔpHR1, and DH5α carrying the vector pRK767 or derivatives of this vector with the full length LHR or the LHR fragments F1, F2, or F3**. Data are shown as means ± standard deviation of triplicate independent experiments.

To verify that high heat resistance in *E. coli* is mediated by proteins encoded by the LHR, the heat sensitive *E. coli* AW1.7ΔpHR1 and DH5α were complemented with LHR or fragments of LHR. LHR or LHR fragments were introduced in *E. coli* AW1.7ΔpHR1 and DH5α after cloning into the low-copy vector pRK767. Fragments F1, F2 and F3 encompassed about 6, 3.3, and 8 kbp, respectively. Cloning of the empty plasmid pRK767 served as control and the heat resistance of the resulting derivatives of *E. coli* AW1.7ΔpHR1 and DH5α was compared to the wild type strains (Figure 5). Cloning the low copy number plasmid pRK767 into *E. coli* AW1.7 did not affect the strain's heat resistance (Figures 1, 5). Strains expressing the full length LHR were as heat resistant as *E. coli* AW1.7 while strains with plasmids containing only a portion of the LHR remained heat sensitive (Figure 5). Complementation of *E. coli* AW1.7ΔpHR1 with the plasmid pHR1 did not alter heat resistance of the strain (Bédié et al., 2012 and data not shown), confirming that the loss of the LHR rather than the loss of the plasmid pHR1 are responsible for the heat sensitive phenotype of this strain.

### Genes encoded by the LHR

The LHR codes for 16 putative ORFs (Figure 4A, Table S1): 2 small heat-shock proteins (sHSPs); a Clp protease (Bojer et al., 2013); several hypothetical proteins with predicted transmembrane domains; a putative sodium/hydrogen exchanger; and several peptidases. Figure 4 compares the operons in *E. coli, C. sakazakii*, and *K. pneumonia.* The predicted function and the conserved functional and transmembrane domains of the predicted proteins are shown in Table S1. The conservation of the ORFs among *E. coli, C. sakazakii*, and *K. pneumonia* is remarkable; most ORFs share more than 99% nucleotide identity to the corresponding genes in *E. coli* AW1.7 (Figure 4B). *E. coli* AW1.7 and AW1.3 share 100% nucleotide identity for 10 of the 16 ORFs (Figure 4B). In *E. coli* AW1.7, the strongest predicted promoter was located 63 bp upstream from ORF1. BPROM analysis predicted that the transcription factor OmpR interacts with this promoter. Another putative promoter is located 26 bp upstream from ORF 9 (Figure 4A). One predicted rho-independent terminator was oriented in the same direction as the ORFs and located 177 bp downstream from ORF 16 (Figure 4A).

In all four strains of *E. coli*, the LHR is flanked by mobile elements or putative transposases (Figure 4B and data not shown). Accordingly, the GC content of the island is 61.8%, substantially higher than the *E. coli* average of ~50% (Figure 4A). In *C. sakazakii* and *K. pneumonia*, the LHR is located on plasmids (Bojer et al., 2010; Gajdosova et al., 2011); however, none of the *E. coli* strains in this study possess plasmids larger than 14 kb (data not shown) and the LHR can thus be assumed to be encoded by the chromosome in the strains of *E. coli* analyzed here. The high degree of sequence identity of the LHR in different species of *Enterobacteriaceae*, the presence of mobile genetic elements adjacent to the LHT, and the divergent GC content suggest that the LHR was acquired by lateral gene transfer.

### Presence of LHR in *E. coli* and other pathogenic species

**Figure 6:**
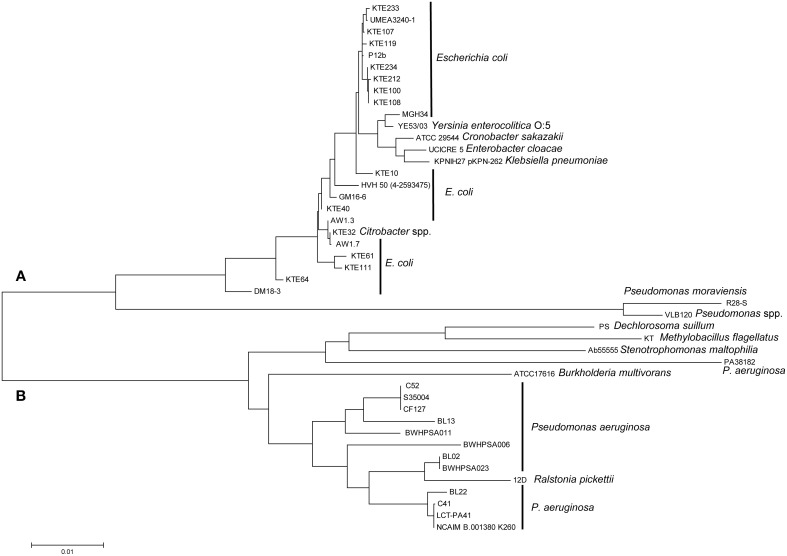
**Maximum-likelihood phylogenetic tree generated from an alignment of LHR sequences (>80% coverage of AW1.7) of disparate species of γ- and β-proteobacteria**. The tree is separated into cluster **(A)**, represented by *Enterobacteriaceae*, and cluster **(B)**, represented primarily by strains of *P. aeruginosa*. Genebank accession numbers of genomes with LHR sequences are indicated in Table S3.

Our study and prior studies with *K. pneumonia* and *C. sakazakii* reported a correlation of the presence of the LHR and heat resistance (Figures 1, 4, 5, Bojer et al., 2010; Gajdosova et al., 2011). The LHR may thus be a marker for heat resistance in *Enterobacteriaceae* and related organisms. To determine the presence of the LHR in bacterial genomes, we performed a BLAST search using the entire LHR, excluding adjacent transposases, against the NCBI Genomes database. This analysis retrieved 41 sequences with more than 80% coverage from several species in the β- and γ-proteobacteria, including pathogenic strains of *Yersinia entercolitica, Enterobacter cloacae, Citrobacter* sp., *Pseudomonas aeruginosa*, and 16 strains of *E. coli*. The sequences were used to calculate a maximum-likelihood phylogenetic tree (Figure 6) that shows remarkable differences from the phylogenetic tree of the bacterial species shown in the tree. The tree is divided into 2 large groups; group A is exclusively composed of sequences γ-proteobacteria (*Enterobacteriaceae* and *Pseudomonas* spp.) while group B includes sequences from β - and γ-proteobacteria (Figure 6).

Group A includes sequences from strains of *E. coli* isolated from urinary tract infections (e.g., KTE#) and food isolates of *E. coli*. The conserved sequence identity between the most distantly related sequences from *E. coli*, DM18.3 and KTE233, is 98.9%, suggesting recent lateral transfer of the LHR. LHR sequences from *E. coli* AW1.3 and P12b, two strains that are phylogenetically closely related, cluster in separate branches of group A while LHR sequences from phylogenetically unrelated strains, e.g., *E. coli* AW1.3 and AW1.7, cluster closely together. LHR sequences from *Yersinia enterocolitica, Enterobacter cloacae, Citrobacter* spp., *K. pneumonia*, and *C. sakazakii* are interspersed with LHR sequences from *E. coli* (Figure 6). The most divergent LHR sequences in group A belong to 2 *Pseudomonas* spp. (Figure 6).

LHR sequences in group B are represented by 13 strains of *Pseudomonas aeruginosa*, including isolates from cystic fibrosis patients. LHR sequences from other pulmonary pathogens include sequences from *Ralstonia pickettii, Burkoholderia multivorans*, and *Stenotrophomonas maltophilia* (Figure 6). *Dechlorosoma suillum* (now *Azospira suillum*; Byrne-Bailey and Coates, 2012) and *Methylobacillus flagellatus* (Chistoserdova et al., 2007) are found in freshwater and sewage and represent the most divergent LHR sequences in group B. The nucleotide identity between the most distant species from group A (*E. coli* KTE233) and group B (*Pseudomonas aeruginosa* NCAIM B.001380 K260) is 87.2% over >80% of the entire LHR sequence. These data provide evidence that the LHR is highly conserved and has been laterally exchanged within the β- and γ-proteobacteria.

To determine the frequency of the LHR in *E. coli* more accurately, we searched the NCBI whole-genome shotgun assemblies (wgs) database in addition to the NCBI Genome database. This analysis retrieved additional LHR sequences predominantly from clinical isolates including UPEC and ETEC (Table 3). Sequences covering >80% of the LHR were identified in 66 out of 3347 strains, with an additional 15 strains found to possess 60–80% of the LHR (Table 3). All sequences are more than 99% identical to the LHR sequence of *E. coli* AW1.7. Including genome sequences obtained in this study, the proportion of LHR-positive strains of *E. coli* is approximately 2% (Table 3).

**Table 3:** **Frequency of LHR in *E. coli.* This table lists *E. coli* genomes or whole genome shotgun sequences containing the locus of heat resistance**.

**Origin of *E. coli* strains sequenced (ref)**	**# of genome sequences**	**# genomes with LHR 80% (60%) coverage^a^**
NCBI genome database^b^	2263	16
Patients with urinary tract infections or bacteremia (1)	317	3 (1)
Clinical isolates of enterotoxigenic *E. coli* (ETEC) (2)	218	13 (4)
*P*atients with urinary tract infections (3)	236	15^c^
Clinical isolates after antibiotic treatment (4)	247	21 (9)
Water isolates of O157:H12 (5)	1	1
ETEC (6)	5	1
Woman with recurrent urinary tract infections (7)	27	3 (1)
Intensive care unit patients (8)	5	2 (0)
Clinical and food isolates (this study)	28	4 (0)
	**Total # of genomes 3347**	**Total LHR 66 (81)**
		**% positive 2.0 (2.4)**

a > 80% coverage and > 95% pairwise nucleotide identity when compared to E. coli AW1.7; values in brackets indicate BLAST hits with 60–80% coverage and > 95% nucleotide identity when compared to E. coli AW1.7.

b Accessed on Aug 11th, 2014.

c 13 of these E. coli strains are included in the NCBI genome database.

*(1) E.coli UTI Bacteremia initiative, Broad Institute (broadinstitute.org) Accessed Aug 11th, 2014; (2) http://genomesonline.org/project?id=16624 Accessed Aug 11th, 2014; (3) http://www.ncbi.nlm.nih.gov/bioproject/193500 Accessed Aug 11th, 2014; (4) http://www.ncbi.nlm.nih.gov/bioproject/233951 Accessed Aug 11th, 2014; (5) http://www.ncbi.nlm.nih.gov/bioproject/PRJNA51127 Accessed Aug 11th, 2014; (6) Sahl et al., 2010; (7); Chen et al., 2013; (8) Hazen et al., 2014*.

### PCR targeting the LHR as a predictor and screening tool for highly heat resistant *E. coli*

**Figure 7:**
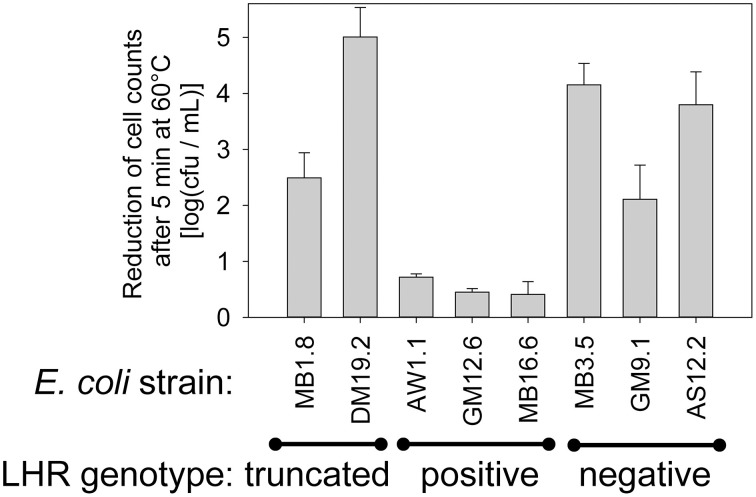
**Correlation of the LHR positive genotype to heat resistance in *E. coli***. Three fragments of the LHR were amplified with PCR (Figure S1) to identify strains with a full length LHR and strains with a full length LHR. Heat resistance of 8 strains of *E. coli* representing 3 LHR-positive and negative strains, respectively, and two strains in which a truncated LHR was detected. Data are shown as means ± standard deviation of triplicate independent experiments.

To determine whether PCR screening for the LHR reliably identifies highly heat resistant strains of *E. coli*, 55 beef isolates of *E. coli* (Dlusskaya et al., 2011) were screened by PCR using primers targeting 3 different regions of the LHR, spanning several ORF's that are unique to highly heat resistant *E. coli* (Figure S1). Out of the 55 strains of *E. coli*, 13 strains were positive for all 3 LHR amplicons (Figure S1) and 2 strains were positive for 2 of the 3 LHR fragments (Figure S1). We selected 3 LHR positive, 3 LHR negative and the 2 strains containing a partial LHR for evaluation of heat resistance at 5 min at 60°C (Figure 7). All LHR positive strains were highly heat resistant but the 2 strains containing a truncated LHR and LHR-negative strains were moderately heat resistant (Figure 7C). The results support the hypothesis that the presence of the complete LHR sequence is required for high heat resistance in *E. coli*.

## Discussion

The resistance of food-borne pathogens to thermal intervention mechanisms challenges the food industry and public heath sectors, requiring a better understanding of the frequency, distribution and detection of heat resistance. This study employed comparative genomics to identify a genetic island, the LHR, which provides exceptional heat resistance in *E. coli*. Core-genome phylogenetic analysis and phylogenetic analysis of the LHR support the conclusion that the LHR is transmitted via lateral gene transfer. Transfer of the LHR occurred between diverse species in the β- and γ-proteobacteria, including enteric and pulmonary pathogens. Screening of food isolates yielded a number of LHR positive strains, and demonstrated that the LHT is a suitable target for identifying heat resistant *E. coli*.

### The LHR mediates heat resistance in *Enterobacteriaceae*

Presence of the LHR in *C. sakazakii* and *K. pneumoniae* correlated to heat resistance of the strains (Bojer et al., 2010; Gajdosova et al., 2011). Of the 36 strains of *E. coli* that were analyzed both with respect to heat resistance and the presence of the LHR, all highly resistant strains carried the LHR and all strains carrying the full length LHR were highly heat resistant. Orthologs of 10 of the 16 ORFs are present in moderately resistant and heat sensitive strains, and a truncated LHR provides only moderate heat resistance. However, presence of the full length locus is unique to highly heat resistant *E. coli*. Complementation with the LHR conferred heat resistance to sensitive strains of *E. coli* only if the entire genomic island was cloned. Heat resistance of *E. coli* is thus dependent on the entire genomic island, and not on the function of a single protein.

The LHR comprises ORFs that are predicted to encode proteins with putative functions in cell envelope maintenance, turnover of misfolded proteins, and heat shock. The predicted products of 5 ORFs possess highly conserved functional domains, including sHSPs (Han et al., 2008) and several proteases. Eight ORFs contain predicted transmembrane domains, including Orfs8-10 and the proteases Orf15 and Orf16. One putative gene, *orf13*, is predicted to encode a sodium/hydrogen antiporter, which corresponds to the interplay of osmotic and heat stress in strains expressing the LHR (Pleitner et al., 2012; Orieskova et al., 2013). Orf16, a predicted membrane protease, possesses a similar domain structure to DegS, a protease involved in the activation of the σ^*E*^ stress pathway in *E. coli* (Alba and Gross, 2004). DegS types of proteases are members of the HtrA (high temperature requirement A) family of proteins, which play a role in protein turnover in the periplasm and are induced by heat shock (Kim and Kim, 2005).

The expression of *orf3*, designated as a Clp protease ClpK, increased heat resistance in *E. coli* DH5α; however, transfer of the entire LHR was required for heat resistance in a *clpP* mutant strain (Bojer et al., 2013), suggesting an interplay of ClpP and other proteins encoded within the LHR. Heterologous expression of *orf7-orf10* from *C. sakazakii* in *E. coli* also resulted in an increase in thermotolerance (Gajdosova et al., 2011), but the heat resistance of the resulting transgenic strains was substantially lower than the level of resistance that was observed in *E. coli* AW1.7 carrying the entire LHR (Figures 1, 5, 7). Deletion of the LHR substantially reduced the resistance of *C. sakazakii* to heat (Orieskova et al., 2013).

The LHR was suggested to be transcribed as a single poly-cistronic mRNA in *K. pneumonia* and *C. sakazakii* (Gajdosova et al., 2011; Bojer et al., 2013). We identified a strong putative promoter upstream of *orf1* which is conserved in both *K. pneumoniae* and *C. sakazakii*. The promoter was predicted to interact with the OmpR, a transcription factor coordinating gene expression in response to osmotic stress (Mizuno and Mizushima, 1990). The LHR is over-expressed in response to osmotic stress (Riedel and Lehner, 2007), which corresponds to the observation that *E. coli* AW1.7 is resistant to heat only when incubated in growth media containing 1–4% NaCl (Ruan et al., 2011; Pleitner et al., 2012), as well as the observation that deletion of the LHR reduces the tolerance of *C. sakazakii* to osmotic stress (Orieskova et al., 2013). The LHR may thus function in response to osmotic and heat stress and its function may be partially dependent on the extracellular concentration of compatible solutes.

### The LHR is transmitted by lateral gene transfer between β - and γ-proteobacteria

The nucleotide identity of the LHR in the *Enterobacteriaceae* is ~99% and the LHR is consistently flanked by mobile genetic elements. Both imply recent lateral gene transfer. The differences in the phylogenetic relationship between strains *E. coli* AW1.3 and P12b support this notion. Based on core-genome sequences, *E. coli* AW1.3 and P12b are highly related and have a recent ancestor. However, their LHR sequences are much more evolutionarily distant; suggesting the strains independently acquired the LHR. Transfer of large genomic elements is well described for genomic islands encoding virulence factors, for example the LEE (Schmidt, 2010). Comparative genomics analysis of the fish pathogen *Edwardsiella tarda* indicated that the LEE of *E. coli* is also transmitted to other *Enterobacteriaceae* (Nakamura et al., 2013). Genomic islands that are transmitted by lateral gene transfer also possess environmental relevance (Juhas et al., 2009) and provide genes for sugar metabolism (Chouikha et al., 2006) or degradation of aromatic compounds (Gaillard et al., 2006). Acquiring multiple genes that require coordinated expression and protein function, e.g., LEE and LHR, can increase the overall fitness of the species.

Genomic islands do not always encode self-transfer capabilities (Shoemaker et al., 2000) and the LHR is located on the chromosome or on plasmids (Bojer et al., 2010; Gajdosova et al., 2011; this study), which may allow exchange through conjugation. Species carrying the LHR occupy similar environmental niches, such as the gastrointestinal tract (*E. coli, Citrobacter* and *Yersinia*), the urinary tract (UPEC and *Yersinia*), and sewage/fresh water (*Enterobacteriaceae*). Remarkably, transfer of the LHR is not restricted to *Enterobacteriaceae* but includes *Pseudomonas* spp. and β-proteobacteria. The GC content and predicted function of the ORFs do suggest a thermophillic origin of the LHR.

### The LHR is present in approximately 2% of strains of *E. coli*, including food isolates and pathogens

This study, in combination with past studies, has identified 7 LHR-positive and highly heat resistant strains (Dlusskaya et al., 2011; Ruan et al., 2011). None of these strains carry virulence factors; however, bioinformatic analyses revealed that about 2% of all the *E. coli* genome sequences or whole genome shotgun sequences contain the LHR with more than 80% coverage and more than 95% nucleotide identity. All studies on the heat resistance of LHR positive strains of *E. coli, Cronobacter*, and *Klebsiella* confirmed that the full length LHR is a reliable predictor of heat resistance. LHR positive strains of *E. coli* include UPEC and ETEC. Because both the LHR and genes coding for virulence factors are highly mobile, highly heat resistant strains of other pathovars likely also exist. A screening of about 100 strains of STEC has not identified highly heat resistant pathogens (Liu et al., 2015), but screening of 100 strains may not suffice to identify a genetic and physiological trait that is present in about 2% of strains. The identification of the genetic determinants of heat resistance provides a rapid screening tool to identify heat resistant *E. coli* in food or clinical isolates. A broader screening of strains and the assessment of their heat resistance will enable to assess the public health significance of heat resistance in *E. coli*.

This study observed a high frequency of LHR-positive and highly heat resistant strains in beef isolates (Dlusskaya et al., 2011). Beef is an important vector for transmission of STEC (Scallan et al., 2011; USDA, 2012) and highly heat resistant *E. coli* are recovered in high numbers from inoculated beef patties that are cooked medium rare and even survive in burger patties that are cooked “well done,” corresponding to an internal temperature of 71°C (Dlusskaya et al., 2011; Liu et al., 2015). To date, the transmission of STEC was attributed to undercooked meat (Schmidt et al., 2002); however, LHR-positive heat resistant pathogens may additionally contribute to foodborne disease. Because these organisms may survive in beef that is cooked to a core temperature of 71°C, cooking meat to a “well done” stage may not always eliminate all pathogenic *E. coli*.

### Conflict of interest statement

The authors declare that the research was conducted in the absence of any commercial or financial relationships that could be construed as a potential conflict of interest.
