# Supplementary material for: Genetic determinants of heat resistance in Escherichia coli
Source: Front Microbiol. 2015 Sep 9;6:932. doi: 10.3389/fmicb.2015.00932 (PMC4563881; doi:10.3389/fmicb.2015.00932)
Supplement: Supplementary file 1 [file Table1.PDF]

Table S1. Predicted proteins encoded by the LHR of *E. coli* AW1.7

Orf	locus tag[1)]	# of amino acids	Protein Product (Conserved Domains[2)])	Transmembrance Regions Predicted[3)]
1	P12B_RS01580.	93	DNA-binding protein (HTH)	--
2	P12B_RS01575	189	small HspC2 heat shock protein (ACD)	--
3	P12B_RS01570	949	ATP-dependent Clp protease, ClpK (2x Clp-N; 2x AAA+; ClpB_D2-s)	--
4	P12B_RS01565	63	Phospholipase (--)	--
5	P12B_RS01560	228	N-terminal fragment of ATP-dependent metallopeptidase HflB (FtsH_ext; AAA+)	2 - i/o: 7 to 26. o/i: 106 to 124
6	-	47	C-terminal fragment of ATP-dependent metallopeptidase HflB (--)	--
7	P12B_RS01555	152	heat shock protein, Hsp20 (ACD)	--
8	P12B_RS01550	304	hypothetical protein, yfdX family (--)	2 - i/o: 8 to 27. o/i: 202-220
9	P12B_RS01545	295	hypothetical protein, yfdX family (--)	1 - i/o:147 to 171
10	P12B_RS01540	203	hypothetical protein (HdeD)	6 - o/i: 26 to 42, i/o: 49 to 70, o/i: 84 to 100, i/o: 104 to 123, o/i: 144 to 162, i/o: 172 to 190
11	P12B_RS01535	381	hypothetical protein (--)	--
12	P12B_RS01530	146	thioredoxin (TRX)	--
13	P12B_RS01525	571	sodium/hydrogen exchanger (KefB; TrkA_N)	11 - o/i: 4 to 25, i/o: 29 to 48, o/i: 56 to 75, i/o: 85 to 108, o/i: 110 to 129, i/o: 147 to 165, o/i: 179 to 197, i/o: 218 to 237, o/i: 287 to 313, i/o: 354 to 373, o/i: 465 to 484
14	P12B_RS01520	165	phosphate-starvation-inducible E family protein (PsiE)	4 - o/i: 28 to 46; i/o: 67 to 83, o/i: 99 to 120, i/o: 126 to 146
15	P12B_RS01515	321	Zn-dependent protease (--)	3 -o/i: 46 to 69, i/o: 173 to 191, o/i: 197 to 214
16	P12B_RS01510	383	trypsin family protein (Trypsin_2; PDZ-serine protease)	2 - i/o: 19 to 39, o/i: 244 to 269

[1)]Locus tag in the genome of *E. coli* P12b (NC_017663)

[1)]Specific domain hits determined by BLAST analysis: HTH - helix-turn-helix domain; ACD - alpha-crystallin domain; Clp-N - Clp amino terminal domain ; AAA+ - ATPases Associated with a wide variety of cellular Activities ; ClpB_D2 - C-terminal, D2-small domain, of ClpB protein ; FtsH_ext - FtsH extracellular domain; HdeD - uncharacterized protein domain; TRX - thioredoxin family; KefB - Kef-type K+ transport system domain; TrkA_N - TrkA-N domain family found in wide variety of proteins; PsiE - phosphate starvation-inducible E; Trypsin_2 - Trypsin-like peptidase domain; PDZ-serine protease - PDZ domain of trypsin-like serine proteases, such as DegP/HtrA

[2)]Predicted using TMPred (Koffman & Stoffel, 1993). Regions represented by amino acids: i/o = inside to outside helices; o/i = outside to inside helices
