# Supplementary material for: Genetic determinants of heat resistance in Escherichia coli
Source: Front Microbiol. 2015 Sep 9;6:932. doi: 10.3389/fmicb.2015.00932 (PMC4563881; doi:10.3389/fmicb.2015.00932)
Supplement: Supplementary file 2 [file Table2.PDF]

Table S2. NCBI genome sequences used in core genome phylogenetic tree

**Strain[1]**	**NCBI Accession Number**	**Phylogenetic Group**
O7:K1 str. CE10	NC017646	D
IAI39	NC011750	D
SMS-3-5	NC010498	D
O127:H6 str. E2348/69	NC011601	B2
ED1a	NC011745	B2
O83:H1 str. NRG 857C	NC017634	B2
LF82	NC011993	B2
CFT073	NC004431	B2
536	NC008253	B2
IHE3034	NC017628	B2
UTI189	NC007946	B2
UM146	NC017632	B2
APEC O1	NC008563	B2
S88	NC011742	B2
SE15	NC013654	B2
NA114	NC017644	B2
UMN026	NC011751	E
42	NC017626	E
O157:H7 str. EDL933	NC002655	E
O157:H7 str. Sakai	NC002695	E
O55:H7 str. RM12579	NC017656	E
Shigella dysenteriae Sd197	NC007606	
HS	NC009800	A
ATCC8739	NC010468	A
UMNK88	NC017641	A
ETEC H10407	NC017633	A
str. K-12 substr. MG1655	NC000913	A
str. K-12 substr. W3110	NC007779	A
DH1	NC017638	A
BW2952	NC012759	A
P12b	NC017663	A
B str. REL606	NC012967	A
Shigella flexneri 2a str. 301	NC004337	
Shigella sonnei Ss046	NC007384	
Shigella boydii Sb227	NC007613	
O121:H19 str. MT#2	AGTJ01000000	B1
O103:H25 str. CVM9340	AJVQ01000000	B1
APEC 078	NC020163	B1
O111:H- str. 11128	NC013364	B1
O26:H11 str. 11268	NC013361	B1
E24377A	NC009801	B1
O103:H2 str. 12009	NC013353	B1
O45:H2 str. 03-EN-705	AGTK01000000	B1
SE11	NC011415	B1
KO11FL	NC016902	B1
IAI1	NC011741	B1
55989	NC011748	B1
O104:H4 str. 2011C-3493	NC018658	B1

[1]All strains are *E. coli* unless noted
