# Supplementary material for: Genetic determinants of heat resistance in Escherichia coli
Source: Front Microbiol. 2015 Sep 9;6:932. doi: 10.3389/fmicb.2015.00932 (PMC4563881; doi:10.3389/fmicb.2015.00932)
Supplement: Supplementary file 3 [file Table3.PDF]

Table S3. Bacterial strains with sequences used for generating the LHR phylogenetic tree

**Strains**	**NCBI Accesion Numbers**
Citrobacter sp. KTE32	NZ_ASQL00000000
Enterobacter cloacae UCICRE 5	AYIK00000000
Escherichia coli KTE40	ASUE00000000
E. coli KTE111	ANUX00000000
E. coli KTE212	NZ_ANTO00000000
E. coli KTE107	ASVI00000000
E. coli KTE234	ANTX00000000
E. coli P12b	NC_017663
E. coli KTE108	ASVJ00000000
E. coli KTE100	NZ_ASVF00000000
E. coli KTE119	NZ_ANUZ00000000
E. coli KTE10	NZ_ANSQ00000000
E. coli KTE64	ASUV00000000
Enterobacter sp. MGH 34	NZ_AYJA00000000
E. coli HVH 50	AYHF00000000
Pseudomonas aeruginosa BL22	AXPE00000000
P. aeruginosa NCAIM B.001380	JMKR00000000
P. aeruginosa C41	NZ_AXOS00000000
P. aeruginosa LCT-PA41	ATJU00000000
P. aeruginosa BWHPSA006	AXQW00000000
P. aeruginosa CF127	AXRO00000000
P. *aeruginosa* S35004	AXRD00000000
P. aeruginosa BWHPSA011	AXQR00000000
P. aeruginosa C52	AXOP00000000
P. aeruginosa BWHPSA023	AXQF00000000
P. aeruginosa BL02	AXPY00000000
Ralstonia pickettii 12D	NZ_ABDZ01000000-NZ_ABDZ01000044
P. aeruginosa BL13	AXPN00000000
E. coli KTE61	ASUU00000000
Stenotrophomonas maltophilia Ab55555	NZ_ALOG00000000
E. coli UMEA 3240-1	NZ_AWCV00000000
E. coli KTE233	ANTW00000000
P. sp. VLB120	NC_022738
Dechlorosoma suillum PS	NC_016616, NZ_ADDK02000000-NZ_ADDK02000022
Methylobacillus flagellatus KT	CP000284, AADX02000000, AADX02000001-AADX02000038
P. moraviensis R28-S	NZ_AYMZ00000000
P. aeruginosa PA38182	HG530068
Cronobacter sakazakii ATCC 29544	NZ_CP011047
Klebsiella pneumoniae KPNIH27	NZ_CP007731
Yersinia enterocolitica (type O:5) YE53/03	NZ_HF571988
Burkholderia multivorans ATCC 17616	NC_010804, NC_010805, NC_010801, NC_010802
