# Supplementary material for: Genetic determinants of heat resistance in Escherichia coli
Source: Front Microbiol. 2015 Sep 9;6:932. doi: 10.3389/fmicb.2015.00932 (PMC4563881; doi:10.3389/fmicb.2015.00932)
Supplement: Supplementary file 4 [file Presentation1.PDF]

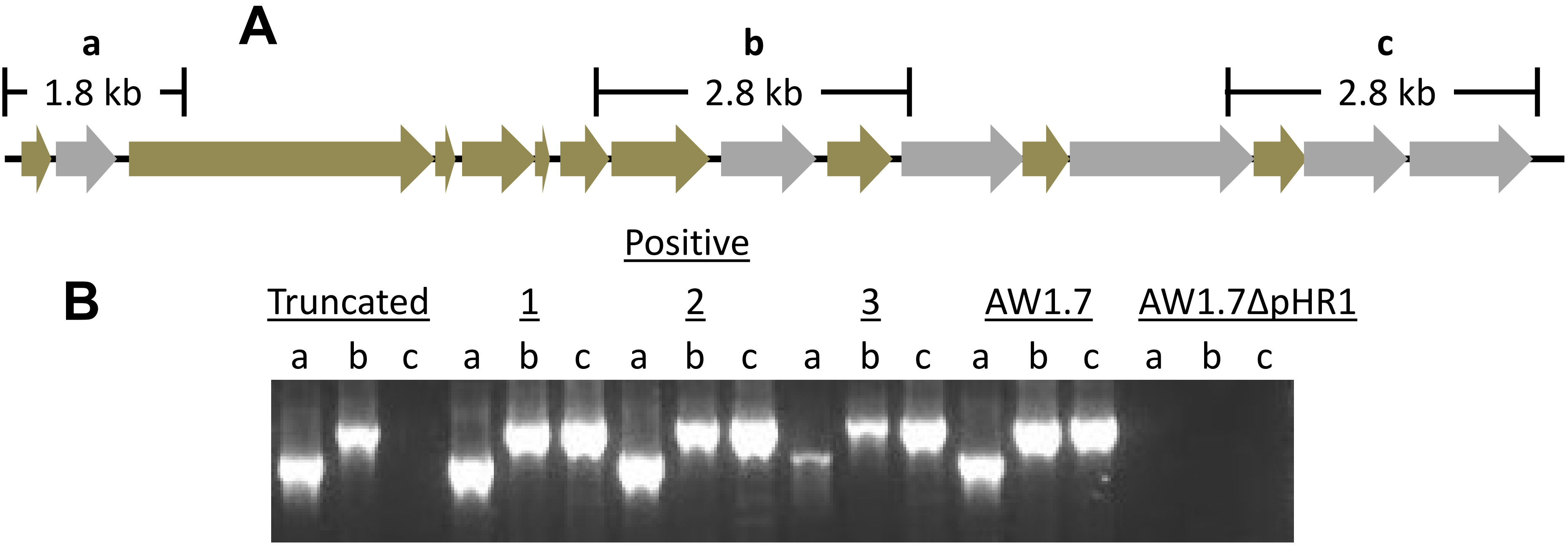

Figure S1. PCR detection of the LHR positive genotype in *E. coli*. Panel A. Representation of the LHR and the fragments that were amplified with primer pairs a, b, and c. **Panel B.** A representative gel image showing amplicons of positive strains and strains with a truncated LHR. E. coli AW1.7 and E. coli AW1.7ΔpHR1 served as positive and negative controls, respectively.
